# A novel approach for assessing neuromodulation using phase-locked information measured with TMS-EEG

**DOI:** 10.1038/s41598-018-36317-z

**Published:** 2019-01-23

**Authors:** Eri Miyauchi, Masayuki Ide, Hirokazu Tachikawa, Kiyotaka Nemoto, Tetsuaki Arai, Masahiro Kawasaki

**Affiliations:** 1 Department of Intelligent Interaction Technology, Graduate School of Systems and Information Engineering, University of Tsukuba, 1-1-1, Tennodai, Tsukuba, Ibaraki 305-8573 Japan; 2 Faculty of Medicine, University of Tsukuba, 1-1-1, Tennodai, Tsukuba, Ibaraki 305-8575 Japan

## Abstract

Neuromodulation therapies such as electroconvulsive therapy (ECT) are used to treat several neuropsychiatric disorders, including major depressive disorder (MDD). Recent work has highlighted the use of combined transcranial magnetic stimulation and electroencephalography (TMS-EEG) to evaluate the therapeutic effects of neuromodulation; particularly, the phase locking factor (PLF) and phase locking value (PLV) can reportedly assess neuromodulation-induced functional changes in cortical networks. To examine changes in TMS-induced PLV and PLF before and after ECT, and their relationship with depression severity in patients with MDD, TMS-EEG and the Montgomery–Åsberg Depression Rating Scale (MADRS; depression severity) were implemented before and after ECT in 10 patients with MDD. Single-pulse TMS was applied to the visual and motor areas to induce phase propagation in the visuo-motor network at rest. Functional changes were assessed using PLF and PLV data. Pre-ECT TMS-induced alpha band (9–12 Hz) PLV was negatively correlated with depression severity, and increments of post-ECT from pre-ECT TMS-induced alpha band PLV were positively correlated with the reduction in depression severity. Moreover, we found a negative correlation between pre-ECT TMS-induced PLF at TMS-destination and depression severity. Finally, differences in post-ECT TMS-induced PLF peak latencies between visual and motor areas were positively correlated with depression severity. TMS-EEG-based PLV and PLF may be used to assess the therapeutic effects of neuromodulation and depressive states, respectively. Furthermore, our results provide new insights about the neural mechanisms of ECT and depression.

## Introduction

Neuromodulatory techniques, such as repetitive transcranial magnetic stimulation (rTMS), transcranial direct current stimulation (tDCS), and electroconvulsive therapy (ECT)^1^, have been used for the treatment of several neurological and psychiatric disorders, including Parkinson’s disease^2^, schizophrenia^3^, and major depressive disorder (MDD)^4,5^. Although the exact mechanisms of these techniques are not yet fully understood, it is thought that neuromodulation regulates functional disturbances in relevant distributed neural circuits^6^ by inducing electric currents that lead to plastic reorganisation of cortical circuits^7^. ECT has long been considered to be highly effective for treating depression^8,9^, which has been associated with abnormal functional connectivity^10^. Some studies have argued that ECT modulates the EEG complexity^11,12^ and the functional connectivity in functional magnetic resonance imaging^13,14^. However, the neural mechanisms of both ECT and depression remain unclear. It is thus important to evaluate the therapeutic effects of neuromodulation using precise methods that capture neuromodulation-induced functional changes.

In recent years, the combination of TMS with electroencephalography (EEG) (hereafter called TMS-EEG) has been used to evaluate the electrophysiological effects of neuromodulatory techniques. Application of TMS pulses enables direct perturbation of cortical regions, and simultaneous EEG recording allows the immediate electrophysiological responses of the stimulated neurons to be measured^15,16^. In particular, TMS is considered as an event or a sensory stimulus for conventional event-related potential paradigms in EEG experiments^17^. Thus, unlike experiments with EEG alone, TMS-EEG allows changes in input parameters and/or stimulation to be closely controlled, which means that more accurate evaluations can be made.

TMS-EEG paradigms have been employed to measure the effect of neuromodulation on the cortex by quantifying changes in motor evoked potentials (MEPs) elicited from the motor cortex^17^ and TMS-evoked potentials (TEPs) elicited from non-motor cortical regions^16^. For example, changes in MEP amplitudes in response to single-pulse TMS of controlled intensity are thought to provide an index of rTMS-induced neuroplasticity of the motor cortex^17–19^. Although MEPs can be easily recorded and analysed^20^, these are influenced by non-cortical confounds such as spinal cord excitability^16^. Moreover, because the exact neural mechanism of neuromodulation is not yet fully understood, it is not clear whether MEP changes accurately monitor neuromodulation-induced neuroplasticity.

On the other hand, TEP-based measures are not influenced by non-cortical confounds^16^ and can be used to analyse the whole brain effect of neuromodulation. For example, analysis of TEP amplitudes allows local responses to neuromodulation to be assessed^21^, and calculations of coherence from TEPs allow global neuromodulation-induced changes to be assessed^22^. However, analysing amplitudes is not an ideal approach to assess the effects of neuromodulation therapies, because these are primarily used to treat psychiatric patients who already exhibit large individual differences in brain activity^10,23^; furthermore, this approach requires a baseline normalisation prior to analysis. Instead, phase, which is calculated by time-frequency analysis, does not require normalisation and can uniformly assess the temporal dynamics of TMS-induced brain activity. Therefore, this approach can be used to assess network connectivity of not only local synchronisation under a single electrode, but also global synchronisation between two distant electrodes, and with a high temporal resolution.

In recent years, it has been suggested that TMS-induced oscillations reflect phase resetting of on-going cortical oscillations^24^. Recent TMS-EEG studies have suggested that single-pulse TMS can induce transient neural oscillations in several frequency bands in different cortical areas of the human brain^25–28^. Kawasaki *et al*.^24^ demonstrated the effect of single-pulse TMS-induced phase resetting across the brain by calculating the phase locking factor (PLF), which is an index of phase-locking of the oscillatory activity on an electrode across trials^29^. Their approach allowed the intensity of information flow through a cortical network to be defined, as well as the causal and directional flow of information, which was identified to flow from visual to motor areas^24^. In addition, Miyauchi *et al*.^30^ found that phase locking value (PLV), a measure of the intensity of phase synchronies between two electrodes^31^, can be used to evaluate the propagation effect of single-pulse TMS-induced phase resetting within a functional network relevant with working memory.

The purpose of this study was to explore the relationship between the TMS-induced changes in PLV and PLF and clinical changes of depression induced by ECT in patients with MDD. This study has focused on the visuo-motor network as the stimulation target because the directionality of information flow in the network has been defined^24^ and the visuo-motor connectivity has been reported to reflect the decrement in visuo-motor performance in depression^32^. Therefore, TMS was applied to visual and motor areas (i.e. targeting the visuo-motor network) to induce phase propagation before and after ECT. Functional changes were calculated using PLF and PLV measures, and ECT-induced clinical changes were measured using the Montgomery–Åsberg Depression Rating Scale (MADRS)^33^.

## Materials and Methods

### Participants

Ten patients (five men and five women aged 27–77 years; mean age: 54.5 years) with MDD completed all experiments. Diagnoses of major depressive disorder were established according to the Diagnostic and Statistical Manual of Mental Disorders (5th edition)^34^ criteria by experienced psychiatrists. Disease severity was evaluated using MADRS within 1 day prior to the first ECT session and 1 day after the ECT session. Psychiatrists interviewed each patient for scoring MADRS. Disease severity was evaluated using the MADRS within 1 day prior to the first ECT session and 1 day after the ECT session. Moreover, dementia was evaluated using the Mini-Mental State Examination (MMSE)^35^. Patients were being treated with antidepressants, antipsychotics, anticonvulsants, and/or benzodiazepines. During the experiments, antidepressant treatments were not altered, but anti-psychotic intake was altered in one patient and benzodiazepines were altered in two patients. Eight patients were ECT-naïve and two patients (Patients 3 and 8) were administered ECT for maintenance. All patients gave written informed consent before participation. The study was approved by the Faculty of Medicine and Medical Sciences, Research Ethics Committee of the University of Tsukuba and was in accordance with the Declaration of Helsinki.

### ECT procedure

Brief pulse, constant current, square wave ECT (Thymatron System IV, Somatics Inc., Lake Bluff, IL, USA) was administrated two or three times a week with bilateral electrode placement. The half-age method^36^ was used for determining energy intensity of the first session, and intensity was then increased by 10–20% in each subsequent session to elicit adequate seizures. Adequate seizures with 100% energy intensity could not be elicited in one patient (Patient 5), and so a sine-wave device (CS-1, SAKAI Medical Co., Ltd., Tokyo, Japan) was used for ECT (110–120 V, 7 seconds) from the 2nd to 6th sessions. Anaesthesia was induced by thiamylal, and succinylcholine was administrated for muscle relaxation. Nicardipine was administrated to prevent excessive hypertension. The number of ECT sessions varied among patients according to the severity of the disease or responses to ECT.

### TMS

TMS-EEG was measured within 1 day prior to the first ECT session and 1 day after the ECT session. Each participant completed two separate sessions (corresponding to visual-TMS and motor-TMS conditions) before and after ECT. The order of conditions was counterbalanced across participants. Each session consisted of 40 TMS applications. Single-pulse TMS was delivered to the respective brain area at jittered time intervals that ranged from 2.5 to 6.5 s (0.5 s steps) and each session lasted for 3 min. TMS sessions were conducted in a dimly lit electronic- and sound-shielded room. During sessions, participants sat in a chair, rested their chin on a chin-rest, and closed their eyes. Participants wore earplugs to help attenuate the effects of TMS-related noise.

TMS was delivered through a figure-of-eight coil with a 70-mm wing diameter that was connected to a biphasic stimulator (Magstim Rapid MRS1000/50, Magstim Company Ltd., UK). We used the flexible arm of a camera stand to fix the coil at the same position and direction for the duration of each session. TMS intensity was fixed to 0.79 Tesla. This intensity was close to 95% of the averaged motor threshold across participants found in our previous study^24^. When delivering TMS to the visual areas, we fixed the TMS coil over the occipital pole (over the Oz electrode) with the handle oriented upward. In contrast, when delivering TMS to the motor areas, we placed the TMS coil tangential to the scalp, with the handle pointing 45° posterolaterally over the central left area (over the C3 electrode).

### EEG recording

Continuous EEG was recorded from 27 scalp electrodes (Ag/AgCl) embedded in a TMS-compatible electrode cap (EasyCap; EASYCAP GmbH, Herrsching, Germany) in accordance with the extended version of the International 10–20 system. EEG data were recorded and amplified using BrainAmp MR+ apparatus (Brain Products, Munich, Germany) at a sampling rate of 1000 Hz. Reference electrodes were placed on the left and the right mastoids and were virtually connected.

We focused on the vicinity of the C3 and Oz electrodes as the left motor and visual areas, because our previous study clearly showed transitions of the TMS-evoked PLF changes from the Oz to the C3 electrodes^24^. Moreover, we stimulated in the vicinity of these areas under the motor and visual TMS conditions. Therefore, this study only examined the synchronisation between motor and visual areas and the information transmission from motor to visual areas or from visual to motor areas.

### EEG pre-processing

EEG data were segmented into 3-s epochs (from 1-s pre-TMS to 2-s post-TMS). To reduce TMS-related artifacts, we removed the EEG data from −1 to 7 ms from TMS onset using linear interpolation, according to previous studies^24,37^. The EEG data were bandpass filtered (0.1–30 Hz). Electrode amplitudes exceeding ±100 µV during the 100 ms before stimulus onset and the 400 ms after onset were considered to indicate the presence of ocular artifacts (i.e. eye movements) and corresponding trials were removed from the analysis.

### EEG analysis

EEG data were analysed using MATLAB software. Time-frequency phases were calculated with wavelet transforms using Morlet’s wavelets function *w*(*t*, *f*0)^29^. Morlet’s wavelets *w*(*t*, *f*0) have a Gaussian shape both in the time domain (SD σ_t_) and in the frequency domain (SD σ_f_) around their central frequency *f*0. The following formula was used to calculate Morlet’s wavelets.

$$\begin{array}{c}w(t,\,f)={({\sigma }_{t}\sqrt{\pi })}^{-\frac{1}{2}}\,\exp (\,-\,{t}^{2}/2{\sigma }_{t}^{2})\,\exp (i2\pi ft)\\ {\rm{with}}\,{\sigma }_{f}=1/(2\pi {\sigma }_{t})\end{array}$$

We used a wavelet that was characterised by a constant ratio (f ⁄σ_f_ = 5), with *f* ranging from 1–20 Hz (1-Hz steps). The time-frequency phases were segmented into the three following frequency bands: theta (4–7 Hz), alpha (8–12 Hz), and beta (13–20 Hz). We used the PLV to identify the TMS-evoked phase synchronisation between visual and motor electrodes. The PLV for an electrode pair (e_*i*_, e_*j*_), time point (*t*), and frequency (*f*) was calculated as follows:

$$PLV(t,\,f,\,{e}_{i},\,{e}_{j})=\frac{1}{N}|\sum _{n=1}^{N}{\exp }^{i(\varphi (t,f,n,{e}_{i})-\varphi (t,f,n,{e}_{j}))}|$$

where ϕ is the instantaneous phase of EEG data and N is the total number of epochs included in the calculation.

In contrast, we used the PLF to identify the TMS-evoked phase resetting at each electrode. The PLF for an electrode (e_*i*_) was calculated as follows:

$$PLF(t,\,f\,,{e}_{i})=\frac{1}{N}\,|\sum _{n=1}^{N}{\exp }^{i\varphi (t,f,n,{e}_{i})}|.$$

### Statistical analysis

Within-group comparisons of MADRS scores (pre- vs. post-ECT) were performed using paired t-tests. Moreover, the relationships between the EEG results (i.e., PLV or PLF) and MADRS scores were analysed using Pearson’s correlation coefficient. We used the statistics toolbox on MATLAB software (R2015b, Mathworks Inc., Natick, MA, USA) to perform all statistical analyses.

## Results

### Clinical effect of ECT

The demographic information of patients is shown in Table 1. All patients had been diagnosed with MDD prior to experiments, and included those with partial and full remission. All patients showed higher MMSE scores than the cut-off point (23/24) for cognitive impairment, which suggests that any individual differences in TMS-EEG results were not due to the effects of cognitive impairment.

**Table 1:** Individual patient demographics.

ID	Age	Sex	Disease	MMSE	MADRS (pre-ECT)	MADRS (post-ECT)	ΔMADRS	No. ECT	medication (dose alteration)
1	77	Female	MDD	25	49	17	32	8	d
2	27	Male	MDD	28	30	5	25	4	d,p,b
3	74	Female	MDD	27	20	5	15	2	d,b
4	54	Female	MDD	29	27	15	12	6	p,b
5	68	Male	MDD	26	24	14	10	6	p (risperidone 1 mg)
6	27	Male	MDD	30	15	9	6	5	p,b (etizolam 0.5 mg↓)
7	43	Male	partial remission	30	6	0	6	6	d, p
8	69	Female	full remission	30	0	0	0	2	d, p,b
9	42	Male	MDD	30	16	17	−1	10	p,b,c (flunitrazepam 1 mg↓)
10	64	Female	MDD, OCD	29	6	8	−2	6	d, b

d: antidepressants, p: antipsychotics, b: benzodiazepines, c: anticonvulsants.

Pre-ECT, five patients (Patients 1–5) showed MADRS scores of over 20, which is cut-off point for moderate depression. Of these, Patient 1 had a score over 35, which is cut-off point for severe depression. In contrast, all patients had post-ECT MADRS scores under 20. Seven patients appeared to benefit from ECT, as indicated by a reduction of MADRS scores post-ECT compared to pre-ECT. While this difference in MADRS scores (pre-ECT vs. post-ECT) was not significant, we nonetheless observed a strong trend for lower MADRS scores after ECT (pre-ECT = (mean ± s.e.m.) 19.50 ± 4.51; post-ECT = 9.00 ± 2.06; paired t-tests, t = 2.075, p < 0.053).

### PLV in visual-TMS conditions

**Figure 1:**
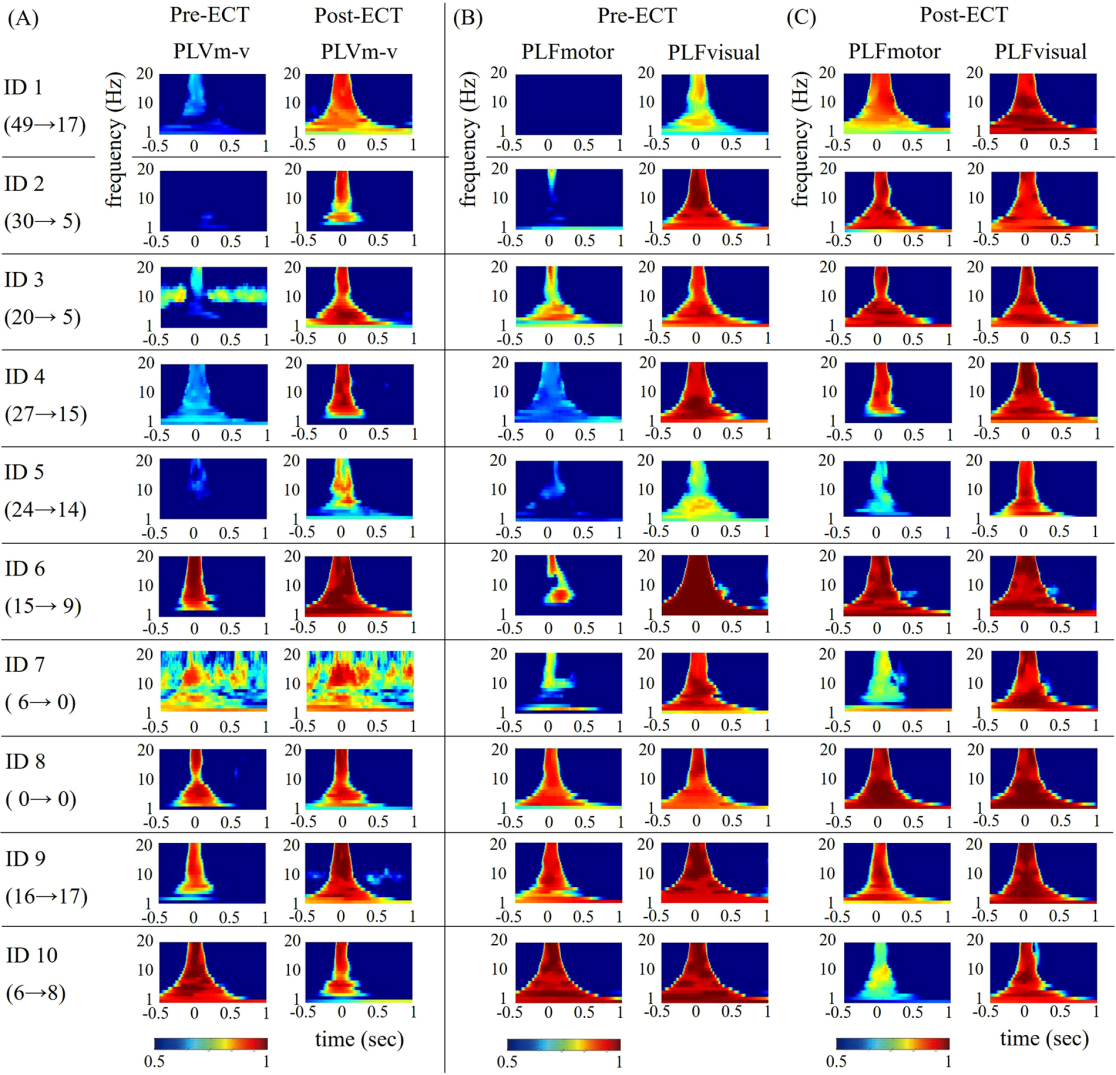
PLV and PLF in visual-TMS conditions. (**A**) Individual time-frequency PLV between motor and visual areas and (**B,C**) time-frequency PLF on motor and visual areas in pre-ECT and post-ECT sessions. The individual MADRS scores at pre-ECT and post-ECT are shown as the left and right numbers below the patient number, respectively.

To measure global synchronisation of the visuo-motor network, we calculated the PLV between visual and motor areas. The individual time-frequency PLVs between motor and visual areas for pre-ECT and post-ECT sessions are shown in Fig. 1A.

Post-ECT, all patients showed transient enhancements of visuo-motor PLVs ranging from 1 to 20 Hz at around TMS onset. These enhancements were observed ahead of TMS onsets because of the wavelet time resolution. Low-frequency PLVs, especially alpha bands (9–12 Hz), increased from TMS onsets.

Pre-ECT, enhancements of PLVs ranging from 1 to 20 Hz were not observed in the five patients with high MADRS scores. In contrast, the five patients with low MADRS scores showed transient enhancements of low-frequency PLV (i.e. alpha PLV) at both pre-ECT and post-ECT.

**Figure 2:**
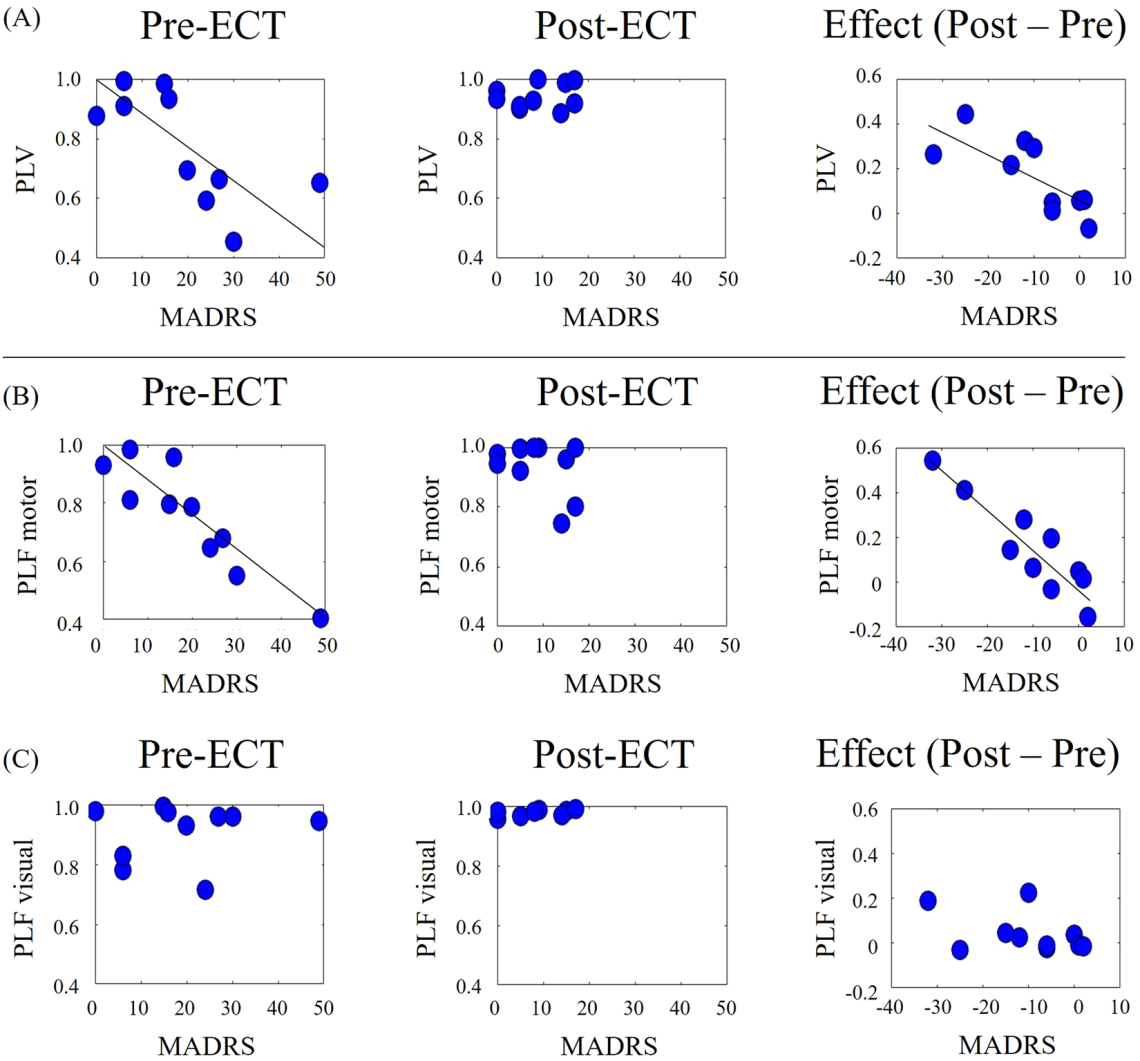
PLV and PLF in visual-TMS conditions. (**A**) Scatter plots between MADRS scores and (**A**) visuo-motor alpha PLV, (**B**) alpha motor PLF, and (**C**) alpha visual PLF, in the pre-ECT (left) and post-ECT (centre) sessions. Scatter plots shown in panels on the right show the differences in MADRS scores and differences in PLV/PLF between pre- and post-ECT.

Next, we calculated the maximum alpha PLVs within a latency window of −500 ms to 500 ms from TMS onset. These were significantly and negatively correlated with MADRS scores at pre-ECT (r = −0.704, p < 0.024), but not at post-ECT (r = 0.182, p = 0.615; Fig. 2A). This correlation was still significant after excluding the three patients (ID:5, 6, 9) whose drugs were altered during the experiments (r = −0.757, p < 0.048). Moreover, the differences in alpha PLVs between pre- and post-ECT were significantly and negatively correlated with differences in MADRS scores between the pre- and post-ECT (Fig. 2A; r = −0.782, p < 0.007). This correlation was still significant after excluding the three patients (ID:5, 6, 9) whose drugs were altered during the experiments (r = −0.808, p < 0.028).

### PLF in visual-TMS conditions

We used the visual (TMS-targeted area) and motor (TMS-distant area) PLFs to measure local synchronisation ability and network-mediated local synchronisation ability (i.e., transmission intensity), respectively. The individual time-frequency PLFs on motor and visual areas at pre-ECT and post-ECT in visual-TMS conditions are shown in Fig. 1B,C. All patients showed transient enhancements of PLFs of visual areas that ranged from 1 to 20 Hz, especially in alpha bands, at around the onset of the TMS at both pre- and post-ECT. These enhancements were particularly strong at post-ECT.

In contrast, the PLFs of motor areas showed no or small alpha transient enhancements in patients with high pre-ECT MADRS scores. However, patients with low pre-ECT MADRS scores showed high alpha PLF enhancements of motor areas. Post-ECT, most patients (all but Patient 5) showed alpha PLF enhancements of motor as well as visual areas.

The maximum values of the alpha PLF (within the latency window −500 ms to 500 ms from TMS onset) of motor areas were significantly and negatively correlated with MADRS scores at pre-ECT (r = −0.907, p < 0.001), but not at post-ECT (r = 0.086, p = 0.813). This correlation was still significant after excluding the three patients (ID:5, 6, 9) whose drugs were altered during the experiments (r = −0.951, p < 0.001). In contrast, the maximum values of alpha PLFs of visual areas were not correlated with MADRS scores at either pre-ECT (r = −0.527, p = 0.118) or post-ECT (r = 0.549, p = 0.100). Individual motor and visual PLF data are shown in Fig. 2B,C. Moreover, the differences in alpha motor PLFs between the pre- and post-ECT were significantly and negatively correlated with differences in MADRS scores between pre- and post-ECT (Fig. 2B; r = −0.911, p < 0.001). No such correlation was observed for alpha visual PLFs (r = −0.427, p = 0.218).

**Figure 3:**
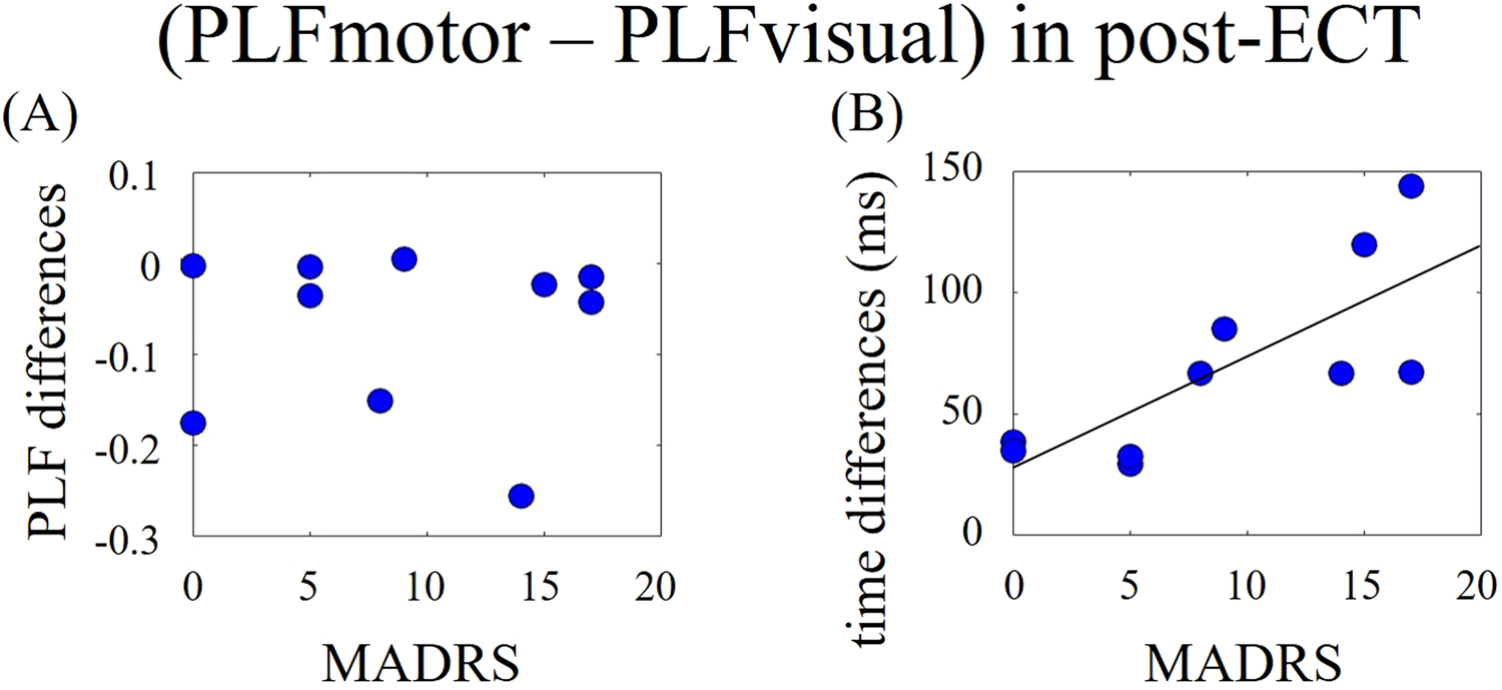
Visual-TMS conditions. (**A**) Scatter plots between MADRS scores and PLF values between visual and motor areas post-ECT. (**B**) Scatter plots between MADRS scores and differences in PLF peak latencies between visual and motor areas post-ECT.

The above results suggested that information transfers from the visual to motor areas post-ECT, but not pre-ECT. Therefore, we calculated the differences in maximum PLF values and their peak latencies from TMS onset between visual and motor areas. We considered the differences in peak latencies of maximum TMS-induced PLF appearances between visual and motor areas to reflect the speed of information transmission within the network. Differences in maximum PLF values were not correlated with MADRS scores (Fig. 3A; r = 0.035, p = 0.925). However, the time differences of PLF peak latencies were significantly and positively correlated with MADRS scores (Fig. 3B; r = 0.778, p < 0.008).

### PLV and PLF in motor-TMS conditions

**Figure 4:**
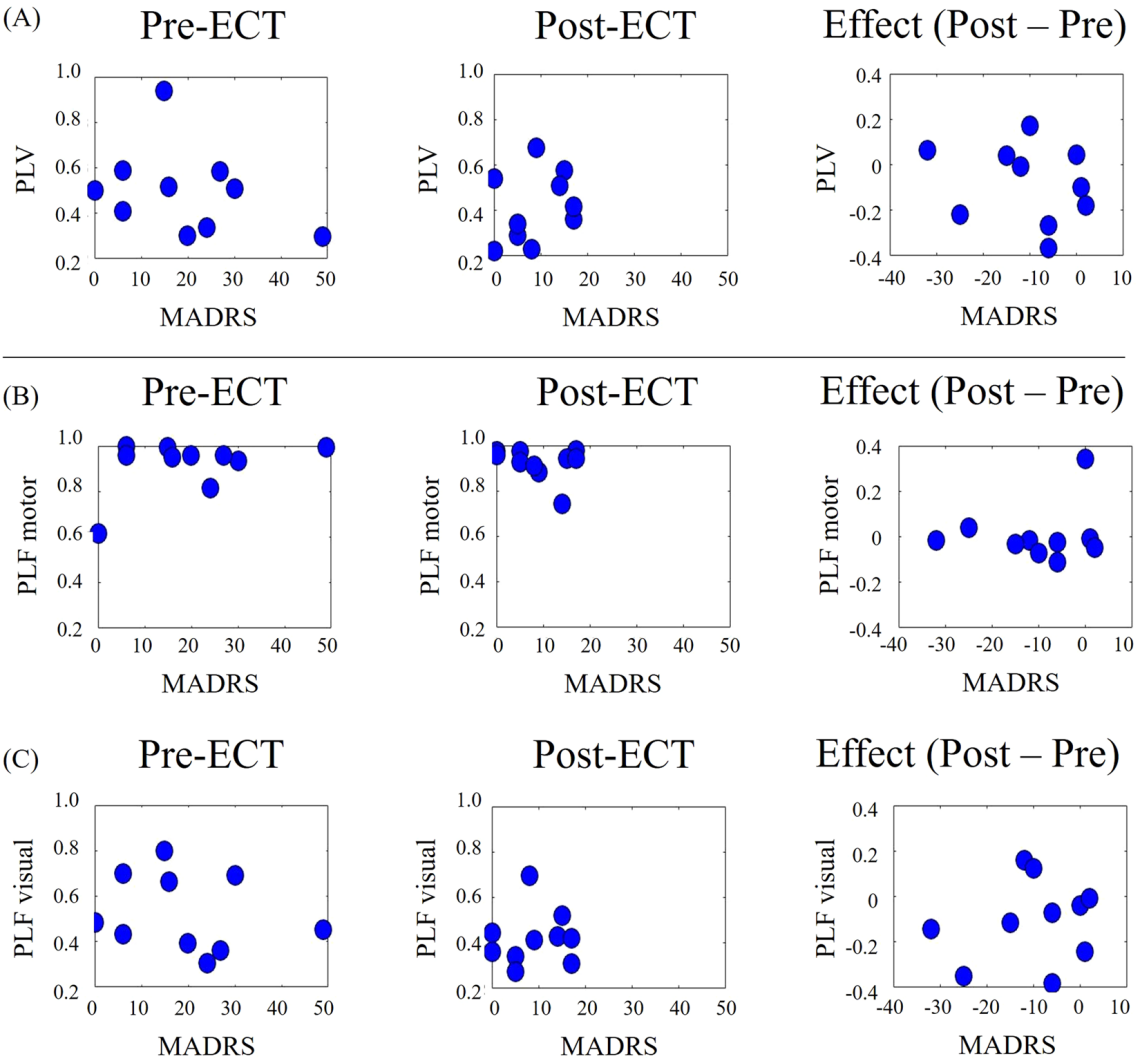
Motor-TMS conditions. (**A**) Scatter plots between MADRS scores and (**A**) visuo-motor alpha PLV, (**B**) alpha motor PLF, and (**C**) alpha visual PLF, in the pre-ECT (left) and post-ECT (centre) sessions. Scatter plots shown in panels on the right show the differences in MADRS and differences in PLV/PLF between pre- and post-ECT.

We conducted the same PLV and PLF analyses for the motor-TMS condition to investigate their relationship with MADRS scores. Most patients showed no transient enhancements of visuo-motor PLVs ranging from 1 to 20 Hz at around the onset of TMS for either pre- or post-ECT time points. Moreover, there were no correlations between the maximum values of alpha PLV and MADRS scores at either pre-ECT (r = −0.331, p = 0.351) or post-ECT sessions (r = 0.287, p = 0.422; Fig. 4A).

For PLFs of the motor areas (i.e. the TMS-targeted areas), most patients showed transient enhancements ranging from 1 to 20 Hz, especially in alpha bands, at around the onset of TMS at both pre- and post-ECT. However, these enhancements were not correlated with MADRS scores at either pre-ECT (r = 0.397, p = 0.255) or post-ECT (r = −0.291, p = 0.415; Fig. 4B). The PLF of visual areas showed no transient enhancements and no correlation with MADRS scores at either pre-ECT (r = −0.216, p = 0.550) or post-ECT (r = 0.099, p = 0.786; Fig. 4C).

The differences of alpha PLVs and PLFs between pre- and post-ECT also showed no correlation with the differences in MADRS scores between the pre- and post-ECT (PLV: r = −0.222, p = 0.539; PLF on motor: r = 0.172, p = 0.634; PLF on visual: r = 0.183, p = 0.612).

## Discussion

In this study, we aimed to investigate whether PLV and PLF can be used to assess depression severity and the ECT-induced functional changes in MDD. The intensity of phase synchronies/propagation within the network was evaluated using PLV, and the intensity and direction of information flow was evaluated using PLF. To this end, we computed the following parameters: (1) TMS-induced PLVs between visual and motor areas, (2) TMS-induced PLF at visual and motor areas, (3) differences in transmission intensities of TMS-induced PLF between visual and motor areas, and (4) differences in TMS-induced PLF peak latencies between visual and motor areas. We then analysed whether those parameters differed before and after ECT, and how these changes were associated with the ECT-induced changes in depression severity.

### PLV for ECT/depression assessment and mechanism

The most interesting finding was that pre-ECT TMS-induced PLV was negatively correlated with depression severity, and that pre-post changes in TMS-induced PLV were positively correlated with the reduction in depression severity. This indicates that the pre-post change in TMS-induced PLV can be used to assess the antidepressant effects of ECT, and, more generally, that TMS-induced PLV can be used to evaluate depressive states. Moreover, these correlations were strongest in the alpha band frequency, which suggests that the inter-areal alpha synchrony of the visuo-motor network is decreased during MDD, and increased by ECT. These results agree with previous findings that the larger increase in cortical excitability induced by 10-Hz rTMS predicts a greater reduction in depressive symptoms after therapeutic neuromodulation^38^. Furthermore, our results further support the idea that depression is associated with a decrease in functional network connectivity at resting state^10^. However, it is somewhat surprising that the results were not best described at the theta frequency band, as was found by previous related studies^24,30^. This may be due to the small number of trials and a consequently limited statistical power. Interestingly, the PLV changes were smaller when the severity of depression was moderate to high, which suggests that ECT-induced alterations of the network only occur in more severe cases of depression.

### PLF (transmission intensity) for depression assessment

We found a negative correlation between pre-ECT TMS-induced PLF at the motor area and the severity of depression, which disappeared after ECT. The pre-post difference in the correlation is likely to be related to the change in the severity of depression over the course of the study. The severity of depression improved remarkably in all subjects after ECT; the severity of depression pre-ECT ranged from “symptom-free” to “severe”, whereas post-ECT severities ranged from “symptom-free” to “mild”. Consistent with our PLV results, it can thus be suggested that ECT-induced network alterations are more prominent in patients with moderate or severe symptoms of depression. In addition, the transmission intensities of TMS-induced PLF within target networks is useful to assess moderate or higher depressive states.

### PLF (time difference) for depression assessment

Interestingly, we found that peak differences of post-ECT TMS-induced PLFs between visual and motor areas were positively correlated with the severity of depression. Such peak differences in PLFs were not detected at pre-ECT, i.e., no propagation effect was found. Our analysis of differences in PLF peak latencies is a new approach, and these findings are therefore only preliminary; however, presuming that differences in PLF peak latencies between the two areas indicate the time of propagation in the network, these results suggest that an alteration of the network is associated with a decrease in the speed of information transmission within the network, and that ECT increases this transmission speed. Moreover, this method could be used to evaluate depressive states when there is sufficient propagation within the network.

### PLF change (local) for mechanism of depression

The TMS-induced PLF at the TMS-targeted areas showed no correlation with depression severity, either before or after ECT. This implies that local activities of the visual and motor areas are not relevant to depressive states, and that depression is associated with a global rather than local dysfunction. These conclusions are consistent with previous findings that a dysfunctional network underlies depression rather than a single brain area^10,39^.

### Visuo-motor network

There was no significant increase in TMS-induced PLV and in TMS-induced PLF at the visual area when TMS was targeted to the motor area. Consistent with previous studies^24,30^, these results indicate that information flows from visual to motor areas. Although the present study was not designed to provide new insights about the relationship between visuo-motor functional connectivity and depression, the observed dysfunction in visuo-motor connectivity is potentially relevant to findings of impairments of sensory and motor processes that are reflected by slowed performance in depression^32^. We used the visuo-motor network as the stimulation target because this is the only network in which the directionality of information flow has been defined^24^. Future investigations could target different networks that have a more evident relationship with depression, such as the subgenual cingulate^23^. For this, it would be necessary to identify the flow directionality of the functional network associated with this area before conducting TMS-EEG to target the network.

### Limitations and future work

Firstly, the small sample size and small number of trials meant that it was not possible to validate the current results. For example, changes in network propagation may be limited to those of whom showed a significant reduction in depression severity. Therefore, in future, larger-scale studies will be required for this. Secondly, the present study did not employ a control group for comparison, and so interpretations are limited to patients with MDD. That said, our results are consistent with studies with healthy subjects, which have reported an increase of TMS-induced PLF values at rest^24^ and increased TMS-induced PLV^30^. Furthermore, it is difficult to implement invasive techniques such as ECT in healthy individuals. However, future studies could compare resting data between patients and healthy controls or use non-invasive therapeutic neuromodulations. Finally, we did not use a neuronavigation system or individual motor thresholds for target selection. Thus, it is possible that TMS-targeted locations were not identical across pre-ECT and post-ECT sessions. Some researchers have recommended the use of a navigated TMS to accurately compute the location of coil based on individual cortical surface anatomy^40^, and future studies could consider this. To build an evaluation system using TMS-EEG based PLV and PLF for the therapeutic effects of neuromodulations, future studies should address the above limitations.

## Conclusion

According to our results, pre-post differences in TMS-induced PLV can be used to assess the antidepressant effects of ECT, and TMS-induced PLV can be used to evaluate depressive states. Moreover, our results suggest that transmission intensities and speed of information transmission calculated from TMS-induced PLF can be used to evaluate moderate or higher states of depression. The present findings therefore have a number of important clinical implications. Namely, our results suggest that the TMS-induced PLV and PLF approaches can be used to assess the therapeutic effects of not only ECT, but also other neuromodulation techniques and medications that are used to improve cortical network function. Furthermore, our findings imply that our proposed approach can be used to assess depressive states, not only in patients with MDD, but also those with other neuropsychiatric disorders or any clinical condition involving impaired cortical networks. Future research using these TMS-induced PLV and PLF approaches will therefore help us to better understand the mechanisms underlying neuropsychiatric disorders.
